# Towards integration of palliative care in patients with chronic heart failure and chronic obstructive pulmonary disease: a systematic literature review of European guidelines and pathways

**DOI:** 10.1186/s12904-016-0089-4

**Published:** 2016-02-13

**Authors:** Naouma Siouta, Karen van Beek, Nancy Preston, Jeroen Hasselaar, Sean Hughes, Sheila Payne, Eduardo Garralda, Carlos Centeno, Marlieke van der Eerden, Marieke Groot, Farina Hodiamont, Lukas Radbruch, Csilla Busa, Agnes Csikos, Johan Menten

**Affiliations:** Department of Radiation-Oncology and Palliative Medicine, University Hospital Gasthuisberg, Leuven, Belgium; International Observatory on End of Life Care, Division of Health Research Lancaster University, Lancaster, United Kingdom; Department of Anesthesiology, Pain and Palliative Medicine, Radboud University Nijmegen Medical Centre, Nijmegen, The Netherlands; Department of Palliative Medicine, University of Navarra Hospital, Pamplona, Navarra Spain; Department of Palliative Medicine, University Hospital Bonn, Bonn, Germany; Faculty of Medicine, Institute of Family Medicine, University of Pécs Medical School, Pécs, Hungary

## Abstract

**Background:**

Despite the positive impact of Palliative Care (PC) on the quality of life for patients and their relatives, the implementation of PC in non-cancer health-care delivery in the EU seems scarcely addressed. The aim of this study is to assess guidelines/pathways for integrated PC in patients with advanced Chronic Heart Failure (CHF) and Chronic Obstructive Pulmonary Disease (COPD) in Europe via a systematic literature review.

**Methods:**

Search results were screened by two reviewers. Eligible studies of adult patients with CHF or COPD published between 01/01/1995 and 31/12/2013 in Europe in 6 languages were included. Nine electronic databases were searched, 6 journals were hand-searched and citation tracking was also performed. For the analysis, a narrative synthesis was employed.

**Results:**

The search strategy revealed 26,256 studies without duplicates. From these, 19 studies were included in the review; 17 guidelines and 2 pathways. 18 out of 19 focused on suffering reduction interventions, 13/19 on a holistic approach and 15/19 on discussions of illness prognosis and limitations. The involvement of a PC team was mentioned in 13/19 studies, the assessment of the patients’ goals of care in 12/19 and the advance care planning in 11/19. Only 4/19 studies elaborated on aspects such as grief and bereavement care, 7/19 on treatment in the last hours of life and 8/19 on the continuation of goal adjustment.

**Conclusion:**

The results illustrate that there is a growing awareness for the importance of integrated PC in patients with advanced CHF or COPD. At the same time, however, they signal the need for the development of standardized strategies so that existing barriers are alleviated.

## Background

The World Health Organization’s (WHO) “Global Burden of Disease” study estimates that, since 2002, chronic or non-communicable conditions have accounted for 87 % of deaths in high-income countries. Moreover, the proportion of deaths worldwide due to such conditions is projected to reach 69 % by 2030 [1].

Chronic heart failure (CHF) and Chronic Obstructive Pulmonary Disease (COPD) are two prominent causes of chronic conditions. CHF in particular is the leading cause of death worldwide whereas COPD is projected to rise to the third highest cause by 2030 [2, 3]. In Europe, more specifically, CHF and COPD are responsible for 1.9 and 2.9,000,000 annual deaths, respectively [4, 5].

Palliative Care (PC) is a specialized medical care targeted in patients living with life-threatening conditions. The aim of PC is the promotion of physical and psychosocial health and thus the improvement of the quality of life of such patients and their families. In order for these objectives to be reached, the focus of PC is typically placed on three principal areas: 1) the alleviation or control of symptoms and side effects of either the disease and/or curative treatment, 2) the timely and continuously updated communication of treatment goals between physicians, patients and their families and 3) the efficient psychological, social and spiritual support for both patients and their families throughout the course of the illness trajectory [6].

Owing to its generic definition, PC can in principle be integrated with curative treatment for both malignant and non-malignant disease [6]. Consequently, even though PC mostly began in cancer care in Europe, it is not surprising that awareness for patients with non-malignant disease has increased [7, 8]. Moreover, extant studies have empirically showed that PC practices can significantly improve the quality of life of patients with chronic conditions [9]. Importantly, there is a general acknowledgement that optimal care for patients with advanced stages of CHF and COPD should rest upon an integrated and holistic approach while simultaneously taking into account patients and family needs throughout the course of the illness [8, 10].

Delivery of PC in patients with advanced CHF and COPD, however, is quite challenging because of the following three barriers i) both diseases are associated with complicated trajectories resulting in uncertain prognostication [11, 12], ii) sudden deaths are common making planning difficult [13, 14], iii) since there is usually a variety of treatment options, patients are not typically well informed about their disease and therefore do not participate actively in decision making. Further, this inhibits the discussion of end-of-life issues [15, 16]. With current evidence showing that access to PC is dominated by patients with cancer [17-19], it is conceivable that these barriers have a prominent role on this aspect.

The implementation of PC in both malignant and non-malignant disease is often based on guidelines and/or pathways. G uidelines are defined as systematically developed statements to assist practitioners and patient decisions about appropriate health care for specific clinical circumstances. Care pathways, on the other hand, are defined as complex interventions for the mutual decision making and organisation of care processes for a well-defined group of patients during a specified period. Consequently, existing guidelines and pathways contain valuable information on current practices for PC in patients with non-malignant disease. Additionally, they provide concise answers to two questions that are critical in planning PC for patients with non-malignant disease: a) when should PC initiate and b) how to integrate PC with curative treatment.

The aim of this study is to systematically review guidelines and pathways of integrated PC for people with advanced CHF and COPD in Europe. By doing so, we obtain an overview of the current level of integration of PC in advanced CHF and COPD in Europe while we document and critically evaluate current practices and recommendations. This study is part of the multi-country European project InSup-C that focuses on integration of PC in cancer and chronic disease in Europe (http://www.insup-c.eu/).

## Methods

To date, a unanimously agreed definition of integrated PC does not exist. For the needs of this study the following definition based on consensus of the InSup-C experts has been employed:

“Integrated palliative care involves bringing together administrative, organisational, clinical and service aspects in order to realise continuity of care between all actors involved in the care network of patients receiving palliative care. It aims to achieve quality of life and a well-supported dying process for the patient and the family in collaboration with all the care givers (paid and unpaid)”.

### Search strategy

The search strategy for this review included an electronic search of the following databases: PubMed, Web of Science, The Cochrane Central Register of Controlled Trials (CENTRAL), CINAHL, EMBASE, BNI, AMED, NHS Evidence, and National Guidelines Clearinghouse. The exact search terms and keywords used for the electronic search are available as an electronic supplement to this paper as well as in the InSup-C website www.insup-c.eu. Besides the electronic database search, the search strategy included citation reference and the hand-searching of the following journals: European Journal of Palliative Care, BMJ Supportive & Palliative care, Palliative Medicine, Journal of Pain and Symptom Management, Medicina Paliativa.

Additionally, for the grey literature search we followed two strategies: i) we contacted named individuals within national scientific medical organizations in order to gather information on guidelines and pathways for CHF and COPD and ii) we performed an electronic search in Google (which was translated in the other six languages of the authors participating in this study). In the UK due to the size of the grey literature, we performed an electronic search in the NHS Evidence database.

### Selection criteria

A systematic review of the literature was conducted of guidelines and pathways about integrating PC into standard care for patients with advanced CHF and COPD. In conformance with the objectives of the InSup-C, the present review is confined to the identification of existing guidelines and pathways in Europe. The other selection criteria of the study are presented in the Table 1 and the exclusion criteria in Table 2 below:

**Table 1:** Inclusion Criteria describes the inclusion criteria for the guidelines and pathways of this study

1. Guidelines and pathways for adult patients
2. Guidelines and pathways for CHF and COPD (latest possible versions)
3. European guidelines and pathways.
4. Guidelines and pathways published from 01-01-1995 to 31-12-2013 (with the start date based on the publication of the Calman-Hine report [81])
5. Languages: English, French, German, Dutch, Hungarian and Spanish (the languages of the authors)
6. Guidelines and pathways that fulfilled at least 2 out of 11 IPC criteria (see explanation below and Table 3).

**Table 2:** Exclusion criteria describes the exclusion criteria for the guidelines and pathways of this study

1. Papers on chronic disease in general.
2. End-of-life guidelines and pathways.
3. General palliative care guidelines/pathways.
4. Guidelines and pathways for children.
5. Guidelines/pathways in languages other than the included ones.

The sixth inclusion criterion concerned the completeness of the content of the included guidelines/ pathways with regard to integrated palliative care. In order to measure the level of the integration of the PC content of the studies we employed a widely used tool with 11 criteria based on the study by Emanuel et al. 2004 [20] (Table 3). A consensus in the InSup-C consortium was reached for the determination of the entry level filter (fulfilment of at least two out of 11 criteria) of this tool.

**Table 3:** Integrated Palliative Care (IPC) Criteria describes the eleven criteria of Integrated Palliative Care for the evaluation of the content of the included guidelines and pathways

1. Discussion of illness limitations and prognosis.
2. Recommendations for conducting a whole patient assessment including the patient’s physical, social, psychological, and spiritual issues, their family and community setting.
3. Recommendations for when to make these assessments
4. Recommendations on when PC should be integrated-referral criteria.
5. Assessment of the patient’s goals for care.
6. Continuous goal adjustment as the illness and the person’s disease progresses.
7. Palliative care interventions to reduce suffering as needed.
8. Advance care planning.
9. Recommendation of involving a PC team.
10. Recommendations on care during the last hours of living.
11. Recommendations on grief and bereavement care.

### Selection procedure

Two authors (NS and KVB) screened all the English search results based on their title and their abstract. The guidelines and pathways that were in the other included languages were screened and translated by two native speaker researchers. Subsequently, NS and KVB sourced and reviewed the translated full texts based on the inclusion criteria and exclusion criteria. Disagreements were reconciled by either consensus or by open discussions in the InSup-C project meetings.

### Data extraction

An extraction form based on the study by Hawker et. al (2002), and it was modified towards the project goals was used to examine included papers [21]. The first two authors extracted data from English guidelines/pathways independently and then cross-checked the results. The same procedure was followed by two native speaker researchers for the non-English ones. Discrepancies were resolved by consensus.

### Data synthesis

Due to heterogeneity of the results, a narrative synthesis was deemed more appropriate and guidelines are presented in Table 4 and pathways in Table 5, while an overall synthesis is presented in Table 6.

**Table 4:** Quality assessment of the Evidence describes the four different categories of the quality assessment of the included guidelines and pathways of this study

High Quality Evidence	Medium Quality Evidence	Low Quality Evidence	Very Low Quality Evidence
Guidelines/pathways based on both systematic reviews and consensus methods or those developed following the NICE protocol [82].	Guidelines/pathways based on systematic review only or based on other types of well referenced evidence.	Guidelines/pathways based on consensus methods only.	Guidelines/pathways that are unclear (e.g. apparently evidence based but failing to clarify how this was obtained).

**Table 5:** Characteristics of included guidelines

Title/Country/Year	Disease	Setting	Integrated Palliative Care Criteria (IPC)	Quality of Evidence
Multidisciplinary guideline Heart Failure/The Netherlands/2010 [22].	Heart Failure	inpatient/outpatient	9 IPC: Discussion of illness limitations and prognosis, Holistic assessments, Timing of PC introduction, Patient’s goals, Continuous goal adjustment, Suffering reduction, ACP*, Involvement of PC team, Last hours of living care.	High
Guideline Palliative care for people with COPD/The Netherlands/ 2011 [23].	COPD	inpatient/outpatient	8 IPC : Discussion of illness limitations and prognosis, Holistic assessments, Timing of holistic assessments, Timing of PC introduction, Patient’s goals, Suffering reduction, ACP, Involvement of PC team.	Medium
Guideline COPD/ The Netherlands/ 2010 [24].	COPD	inpatient/outpatient	5 IPC: Discussion of illness limitations and prognosis, Timing of PC introduction, Patient’s goals, Continuous goal adjustment, Suffering reduction.	Low
Guideline Heart failure/The Netherlands/2010 [25].	Heart failure	inpatient/outpatient	7 IPC: Discussion of illness limitations and prognosis, Holistic assessments, Timing of PC introduction, Patient’s goals, Suffering reduction, Involvement of PC team, Last hours of living care.	Low
Multidisciplinary guideline diagnostics and treatment of COPD/ The Netherlands/ 2010 [26].	COPD	inpatient/outpatient	5 IPC : Discussion of illness limitations and prognosis, Holistic assessments, Timing of PC introduction, Patient’s goals, Suffering reduction.	High
95 Management of chronic heart failure. A national clinical guideline/UK- Scotland/2007 [27].	Heart failure	inpatient/outpatient	4 IPC: Discussion of illness limitations and prognosis, Timing of holistic assessments, Patient’s goals, Suffering reduction.	High
Living and dying with advanced heart failure: a palliative care approach/UK- Scotland/2008 [28].	Heart Failure	inpatient/outpatient	10 IPC: Discussion of illness limitations and prognosis, Holistic assessments, Timing of holistic assessments, Timing of PC introduction, Patient’s goals, Continuous goal adjustment, Suffering reduction, ACP, Involvement of PC team, Last hours of living care.	High
NICE clinical guideline 101: Management of chronic obstructive pulmonary disease in adults in primary and secondary care/UK/2010 [29].	COPD	inpatient/outpatient	3 IPC: Holistic assessments, Suffering reduction, Involvement of PC team.	High
Chronic Obstructive Pulmonary Disease Services/UK- Scotland/ 2010 [30].	COPD	inpatient/outpatient	5 IPC: Discussion of illness limitations and prognosis, Holistic assessments, , Patient’s goals, ACP, Involvement of PC team.	High
Global Strategy for Diagnosis, Management, and Prevention of COPD/UK/ 2013 [31].	COPD	inpatient/outpatient	7 IPC: Discussion of illness limitations and prognosis, Timing of holistic assessments, Timing of PC introduction, Continuous goal adjustment, Suffering reduction, ACP, Involvement of PC team.	High
Heart Disease: quick reference guide/UK/ 2012 [32].	Heart Failure	inpatient/outpatient	3 IPC: Discussion of illness limitations and prognosis, Timing of PC introduction, Suffering reduction.	Low
IMPRESS guide for commissioners on supportive and end of life care for people with COPD/UK/ 2012 [33].	COPD	inpatient/outpatient	9 IPC : Discussion of illness limitations and prognosis, Holistic assessments, Timing of PC introduction, Patient’s goals, Continuous goal adjustment, Suffering reduction, ACP, Involvement of PC team, Grief and bereavement care.	High
Services for people with chronic obstructive pulmonary disease CMG43/UK/ 2011 [34].	COPD	inpatient/outpatient	11 IPC: Discussion of illness limitations and prognosis, Holistic assessments, Timing of holistic assessments, Timing of PC introduction, Patient’s goals, Continuous goal adjustment, Suffering reduction, ACP, Involvement of PC team, Last hours of living care, Grief and bereavement care.	High
Services for people with chronic heart failure/UK/2011 [35].	Heart Failure	inpatient/outpatient	9 IPC : Discussion of illness limitations and prognosis, Holistic assessments, Timing of holistic assessments, Timing of PC introduction, Suffering reduction, ACP, Involvement of PC team, Last hours of living care, Grief and bereavement care.	High
Chronic obstructive pulmonary disease: Management of chronic obstructive pulmonary disease in adults in primary and secondary care/UK/ 2010 [36].	COPD	inpatient/outpatient	2 IPC: Suffering reduction, Involvement of PC team.	High
Best practice guidance on developing a respiratory service specification/UK/ 2008 [37].	COPD	inpatient/outpatient	2 IPC:, Suffering reduction, Last hours of living care.	Low
ESC Guidelines for the diagnosis and treatment of acute and chronic heart failure 2012/Europe/2012 [38].	Heart Failure	Acute setting	8 IPC: Discussion of illness limitations and prognosis, Holistic assessments, Timing of holistic assessments, Timing of PC introduction, Continuous goal adjustment, Suffering reduction, ACP, Involvement of PC team.	High

The included guidelines are described in different categories: title, country and year, type of disease, setting, Integrated Palliative Care (ICP) criteria and quality of evidence. *ACP*= Advance Care Planning, *COPD*= Chronic Obstructive Pulmonary Disorder.

**Table 6:** Characteristics of included pathways

Title/Country/Year	Disease	Setting	Integrated Palliative Care Criteria (IPC)	Quality of Evidence
Consensus on Integrated Care for Disease Exacerbations of COPD. (ATINA-EPOC)/Spain/2012 [39].	COPD	-	8 IPC: Discussion of illness limitations and prognosis, Holistic assessments, Timing of holistic assessments, Timing of PC introduction, Patient’s goals, Continuous goal adjustment, Suffering reduction, ACP*.	Low
End of life care in heart failure: A framework for implementation/ UK/ 2010 [40].	Heart Failure	inpatient/outpatient	7 IPC: Holistic assessments, Patient’s goals, Suffering reduction, ACP, Involvement of PC team, Last hours of living care, Grief and bereavement care.	Low

the included pathways are described in different categories: title, country and year, type of disease, setting, Integrated Palliative Care (ICP) criteria and quality of evidence. *ACP*= Advance Care Planning, EoL=End-of-Life, *COPD*= Chronic Obstructive Pulmonary Disorder.

### Quality assessment

For the evaluation of the quality of the evidence a four-point Likert scale tool (high quality (4) to very low quality (1)) was developed by the project consortium (Table 4). It is important to highlight that the assessment employed in the present systematic review does not assess the quality of the implementation of the included guidelines/pathways. Rather, it provides a means of evaluating the principles upon which they have been proposed.

## Results

**Fig 1:**
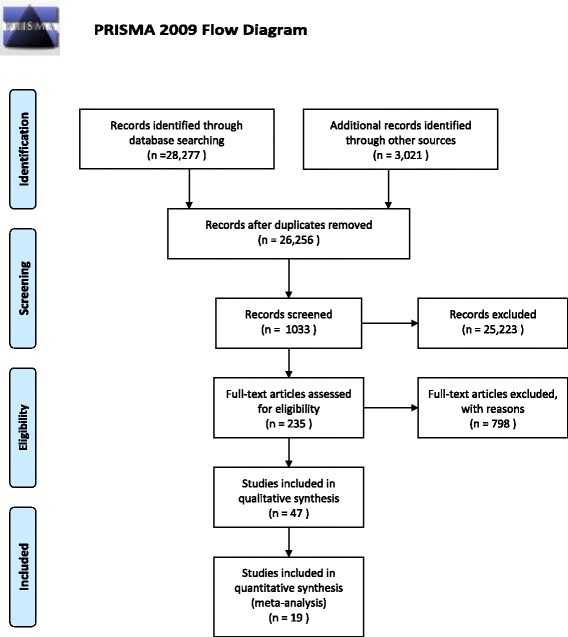
Flow diagram of study selection procedure

We identified a total of 31,298 potentially relevant articles, with 28,277 originating from the electronic database searching and 3021 from the grey literature and the citation tracking. The process of contacting professional experts did not return any further result. After the exclusion of the duplicate results we had 26,256 results of which 25,223 were excluded based on their titles or abstracts. From the 1033 remaining results, we identified 235 guidelines/pathways eligible for full-text screening. The final review included 17 guidelines and two pathways (in total 19) [22–40]. The properties of these studies are available in Tables 5 and 6. A flow diagram of the selection procedure and results (using the PRISMA tool [41]) is presented in Fig. 1 below.

Seventeen guidelines and two pathways were included in the study. Of the 17 guidelines included in the final review, eleven originated from UK, five from the Netherlands and one from more than one European country. Of the two pathways, one pathway originated from Spain and one from UK. From these results, eleven guidelines/pathways were concerned with COPD and eight with CHF. A synthesis of the key point recommendations for all the included CHF and COPD guidelines and pathways in relation to the 11 IPC Criteria is presented in Table 7. Moreover, throughout this section results correspond to the combined set of pathways and guidelines.

**Table 7:** Key point recommendations in relation to the IPC criteria provides the key recommendations of Integrated Palliative Care of the included guidelines and pathways

IPC Criteria	References of guidelines and pathways	Key point recommendations
Discussion of illness limitations and prognosis	[22–28, 30–35, 38, 39]	“Open communication between patient and doctor.”
Holistic assessment	[22, 23, 25, 26, 28–30, 33–35, 38–40]	“Address physical, emotional, social and spiritual needs.”
Timing for holistic assessments	[23, 27, 28, 31, 33–35, 38, 39]	“Work closely with clinicians to agree on the indicators for the exact timing of the holistic assessments.”
Timing for PC introduction	[22–26, 28, 31–35, 38, 39]	“Early integration of PC in the disease trajectory.”
Patient’s goals assessments	[22–28, 30, 33, 34, 39, 40]	“Disease specific management plans and care plans should be based around patient’s personal goals.”
Continuous goal adjustment	[22, 24, 28, 31, 33, 34, 38, 39]	“Regular assessment of patients’ PC needs and continuous communication and collaboration between care teams and organizations.”
Suffering reduction	[22–29, 31–41]	“Timely access to symptom control and administration of appropriate medication”
Advance care planning (ACP)	[22, 23, 28, 30, 31, 33–35, 38–40]	“Early discussion of ACP, including patients’ end-of-life needs and preferences.”
Involvement of PC team	[22, 23, 25, 26, 28–31, 33–36, 38, 40]	“Specialist PC is provided by multi-professional PC teams, including physicians, nurse specialists, psychologists, chaplains, social workers, pharmacists and other appropriate allied health professionals.”
Recommendations on care during the last hours of living	[22, 25, 28, 34, 35, 37, 40]	“Care in the last days of life should be available 24 h a day, including rapid access services, symptom control and assessment of end-of-life preferences.”
Grief and bereavement care recommendations	[33–35, 40]	“Provide family bereavement support and ensure there is access to spiritual care and chaplaincy services.”

There was almost unanimous agreement (18/19) that the focus of PC interventions should be placed on reduction of suffering through the provision of appropriate medication and psychological support. Recommendations for discussions about illness prognosis and limitations were found in 15 out of 19 of the included guidelines and pathways.

It was found that the holistic approach, i.e. the assessment of the patient’s physical, psychological, social and spiritual issues, was recommended in 13 out of 19 guidelines/pathways, however, only 8/19 included instructions on when these assessments should take place.

Recommendations concerning the involvement of a PC team were reported in 13/19. All these 13 guidelines and pathways additionally promote the composition of a multidisciplinary PC team that involves professionals from different disciplines e.g. physicians, disease specialists, nurses, psychologists, psychiatrists, chaplains, nutritionists, physiotherapists, etc. Seven guidelines/pathways recommend the involvement of personnel that are additionally trained in PC. On the other hand, the utilization of advance care planning and the assessment of the patients’ goals of care were mentioned in 11/19 and 12/19 guidelines and pathways respectively.

**Fig 2:**
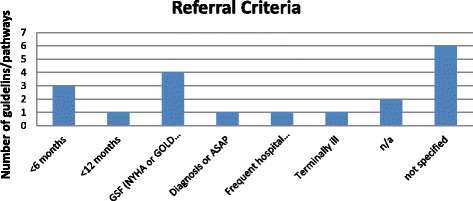
Absolute number of guidelines/pathways in relation to the referral criteria for PC

From the included guidelines/pathways 12 out of 19 discussed explicitly the referral criteria, i.e. the point at which PC should be initiated. However, among those that made an explicit recommendation, the analysis yielded large variations. A minority (4/19) used the specific referral criteria mentioned in the Gold Standards Framework or stages III or IV in the New York Heart Association (NYHA) [42] or stages III and IV in the Global Initiative for Chronic Obstructive Lung Disease (GOLD) [22, 24, 28, 35, 43]. The timing as to when to initiate PC varied with 3/19 saying in the last six months but none of them invoked the surprise question [23, 25, 26]. One guideline reported that PC should be applied in the last 12 months of life [39], whereas another one stated that the referral criteria should be for terminally ill people without however defining the exact timing [40]. One guideline encouraged considering integration of PC from the moment of diagnosis or as soon as possible [27]. Finally, one guideline recommended that the exact PC timing should depend on the frequency of hospital admissions or exacerbations [38]. The referral criteria distribution is presented in Fig. 2.

Only four (4/19) of the included guidelines/pathways elaborate on aspects such as grief and bereavement care (post mortem), seven (7/19) gave recommendations on how to treat the patient in the last hours of life and with continued goal adjustment mentioned in 8/19 of the examined documents.

Using the quality assessment for the evaluation of the evidence (see Table 4), 6/17 guidelines/pathways scored low for quality as they were based on consensus methods only. One guideline was categorised as medium quality; based on systematic review only or based on other types of well referenced evidence. Finally, 12/19 of guidelines/pathways were classified as high quality evidence. According to our findings, the majority of the guidelines/pathways were not devised in collaboration with PC physicians. At this point it is important to highlight that the assessment employed in this review does not assess the quality of the implementation of the included guidelines/pathways. Rather, it provides a means of evaluating the principles upon which they have been proposed.

## Discussion

The majority of the included studies originated from the UK (11 guidelines and one pathway). This is probably due to the fact that PC originated from the UK [44]. The results revealed considerable discrepancies in the integration of PC guidelines, not only in the level of implementation, but in the level of what is conceptually deemed important as well. However, despite such disparities, almost all guidelines/pathways emphasise that the priority of integrated PC should be the reduction of suffering, by effective symptom control. This convergence of opinion is noteworthy for two reasons. First, it accords with what organizations such as the WHO [45], the European Society of Cardiology [46], the European Respiratory Society [47] and others identify as the primary objectives of PC. Second, it conforms to perceptions of both physicians and patients [48, 49]. 

The determination of the referral criteria in the application of PC to patients with advanced CHF or COPD has been a subject of debate [12, 50–54]. This is mainly due to the fact that, as opposed to cancer, illness trajectories of CHF and COPD are quite variable including sequences of deteriorations and (partial) relapses [55]. In fact, even though prognosis of both advanced CHF and COPD is poor [55, 56], prognostication is inexact. Also, the predictive capacity of the various utilized tools is at best moderate and is further reduced by the frequent occurrence of sudden deaths, frequent relapses, comorbidity, and so on. Consequently, physicians are often reluctant to discuss PC options and, interestingly, when they do so, the reaction of patients involves negative surprise; this might also be attributed to the fact that public understanding of these diseases is not linked to dying, unlike for example cancer [57, 58].

In the present study, nearly half of the included guidelines/pathways did clarify referral criteria. However, even among those that provide a recommendation, no appreciable convergence of opinions was observed, as our analysis demonstrated widely diverse referral criteria. Moreover, the appropriateness of many of these referral criteria is questionable because they heavily rely on prognostication; in fact, even the three guidelines/pathways that opted for “last six months” did not based their recommendation on the surprise question but rather on prognostic models. Interestingly, at the same time, guidelines recognise the need for communication between clinicians and patients concerning the limitations of prognostication which appears to lead to a contradictory view. A possible way to resolve this issue was demonstrated by communicating to the patient the potential risks involved in future admissions so paving the way for an advance care planning discussion. Since only one guideline explicitly promoted the early inclusion of PC alongside standard treatment, it is evident that, despite recommendations of medical associations [47], the perception that PC should be primarily concerned with “end-of-life” still prevails [59].

Concerning decision making and advanced care planning, patients with advanced CHF or COPD are quite unlikely to get engaged in discussions concerning treatment options/preferences and end-of-life issues because i) patient-physician communication about end-of-life care might be less likely to occur, ii) it is more complicated to initiate this for patients with less certain prognosis [60–62] and iii) as mentioned above, public understanding of these diseases is not directly linked to dying which can inflict negative reactions from patients [57, 58]. The scarcity of patient-physician discussions concerning treatment options/preferences and the frequent total absence of discussions on end-of-life issues result in less informed patients who are, nevertheless, willing to both familiarize themselves with aspects of their disease and express their preferences [63]; these adverse effects are more apparent nowadays because many patients search for relevant information on the web and can thus challenge or question medical decision making [64].

The majority of the included guidelines/pathways identified the need for enhanced communication for both treatment options/preferences and end-of-life issues and explicitly advocate for it. Nonetheless, the necessary further steps to achieve this, such as advance care planning and continuous goal adjustment are absent from a considerable amount of the included studies. Consequently, since patient-physician communication, advanced care planning and continuous goal adjustment are interlinked, ignoring the latter two might result in suboptimal PC. It is interesting to note that similar findings were reported in a very recent systematic review of integrated PC in guidelines and pathways in cancer, which implies that poor communication is evident independently of the disease trajectory [19].

The holistic approach to care including comprehensive assessment of physical, psychological, spiritual and social needs is backed by robust evidence [65–69]. However, its practical implementation is quite challenging because it is based on the well-orchestrated coordination of and cooperation between different specialties while often requiring the assumption of additional, novel duties from the involved personnel. Current research has documented that these requirements are frequently unmet due to implemented i) the reluctance of physicians to advocate expansion of specialist PC services, ii) the obscurity of the roles of doctors and nurses in different specialties and iii) the limited funding and infrastructures [69, 70]. As a consequence, the same studies hint that the holistic approach is often poorly implemented in practice. In view of these facts, we can infer that a stand-alone recommendation for the employment of the holistic approach is inadequate. Rather, such recommendations should be supplemented with detailed instructions that exemplify and quantify the roles of the involved personnel, their interaction and timing of the assessments. The prominence of the holistic approach is acknowledged by the majority of the included guidelines/pathways [22, 25, 28]. Moreover, they provide specific recommendations for controlling physical symptoms, relieving psychological issues and addressing spiritual needs of the patients.

As regards the composition of the PC team, most of the guidelines/pathways advocate a multidisciplinary approach that involves professionals from different disciplines who are additionally trained in PC [71]. The advantages of the multidisciplinary approach over the uni-disciplinary one have been documented and advocated for multiple times in the literature both for CHF and COPD [17, 45, 72–75]. It is also important to note that combining a holistic approach with a multidisciplinary PC team has been posited to increase the benefits of PC [76]. The advantages of this combination have been recognized by most guidelines and pathways in this study.

Finally, our analysis revealed a lack of emphasis on recommendations on the last hours of life and bereavement care. It is striking that most of the guidelines/pathways identify PC as an end-of-life concept. Both aspects have consistently been identified as significant components of a complete and optimized integrated PC [12, 77]. However, and despite being explicitly promoted [50, 78–80], they are frequently overlooked as is the case with the present guidelines/pathways. Consequently, future guidelines and pathways should increase their focus on these aspects.

### Study limitations

The search strategy employed herein is quite generic in order to cover as many guidelines/pathways related to CHF and COPD as possible which led to a large number of titles being screened. The main reason for employing this search strategy is because we conducted two studies, one focusing on cancer patients (reported elsewhere) and another focusing on CHF and COPD. It is conceivable that a different search strategy would reveal a somewhat different list of results. Still, the employed search strategy is deemed to be general enough to cover the vast majority of the existing CHF and COPD studies. This was supported through citation tracking and reference list checking.

The lack of a standardised and universally accepted definition of integrated PC constitutes a limitation of this study. As a consequence the search strategy uncovered a rather heterogeneous body of working touching on a variety of aspects of integrated PC. Still, the definition employed herein is deemed general enough to encapsulate the most relevant aspects of integrated PC.

A second limitation is linguistic and refers to the restriction to European guidelines/pathways published in Dutch, English, French, German, Hungarian and Spanish. It is quite possible that several guidelines/pathways exist in other European languages as well; our first electronic search returned potential candidates in Italian and Swedish that were excluded for the reasons described above. Further, additional information could have been obtained if we had included studies from other continents as well.

The third limitation of this study pertains to the tool employed for the evaluation of the completeness of the content of the guidelines/pathways. Following consensus between the authors and the experts participating in the InSup-C project, Emmanuel’s criteria were adopted on the basis of their completeness. In fact, the range covered by Emmanuel’s criteria is large enough to ensure an overlap with a potential alternative. Consequently, even though the employment of a different evaluation tool might have provided alternate results, modifications are expected to be minor. It doesn’t however help us know which guidelines work the best in practice.

## Conclusions

We have systematically reviewed the literature for guidelines/pathways of integrating PC in patients with advanced CHF or COPD in Europe. Existing guidelines/pathways thoroughly discuss the aspects like the reduction of suffering, the holistic approach, the enhanced communication and the involvement of multi-disciplinary PC team. However, other related aspects such as referral criteria, advanced care planning, recommendations on the last hours of life and bereavement care are only partially touched or addressed. Moreover, several suggested recommendations and solutions from the guidelines are either insufficiently clear or even at odds with existing directives and well-documented findings. For example, several guidelines/pathways recommended referral that rely on prognostication while at the same time acknowledged the limitation for acquiring an accurate one.

Overall, the results of this systematic study illustrate that there is a growing awareness for the importance of PC in patients with advanced CHF and COPD. At the same time, however, they signal the need for the development of standardized and conceptually unambiguous strategies so that existing barriers are alleviated. In this respect, given that prognostication for both CHF and COPD is difficult, emphasis should be placed on the determination of referral criteria that are independent of it and thus straightforward to realise in practice. Moreover, particular attention should be paid to the communication of end-of-life issues that consistently appears as a bottleneck in PC for patients with advanced CHF and CODP. Further, it is critical that the nearly total absence of discussions concerning end-of-life issues is alleviated so that both the efficacy of PC services and the number of beneficiaries are enhanced. This is instrumental for the improvement of existing PC practices that have been consistently shown to be suboptimal.

### Ethics

Ethical approval was not required for this study. However this study was performed according to the standards of PRISMA guidelines.

### Consent

No consent was required for the conduction of this study.

## Abbreviations

AMED: allied and complementary medicine
BNI: British nursing index
CENTRAL: Cochrane Central Register of Controlled Trials
CHF: chronic heart failure
CINAHL: Cumulative index to nursing and allied health literature
COPD: chronic obstructive pulmonary disease
IPC: integrated palliative care
NHS Evidence: National heath service evidence.
PC: Palliative care
